# Targeting the gut–brain axis: novel strategies for disease intervention from mechanisms to clinical practice

**DOI:** 10.3389/fmicb.2026.1923456

**Published:** 2026-10-07

**Authors:** Fan Lele, Guo Yuying, Liu Jin, Han Shuwen, Zhuang Jing

**Affiliations:** 1 Huzhou Central Hospital, Fifth School of Clinical Medicine of Zhejiang Chinese Medical University, Huzhou, Zhejiang, China; 2 Huzhou Central Hospital, Affiliated Central Hospital Huzhou University, Huzhou, Zhejiang, China; 3 Zhejiang-France United Laboratory of Integrated Traditional Chinese and Modern Medicine in Colorectal Cancer, Huzhou, Zhejiang, China; 4 School of Medicine, Tarim University, Alaer, Xinjiang, China

**Keywords:** bidirectional regulation, disease, gut-brain axis, intervention strategies, microbiota

## Abstract

The gut–brain axis is a bidirectional communication network linking the gastrointestinal tract and the central nervous system (CNS), in which the gut microbiota and its metabolites have emerged as important modulators. Alterations in the gut microbiota have been reported in a range of neurological, psychiatric, and metabolic disorders, but their underlying mechanisms and causal relevance in humans are still unclear. This review examines the major neural, endocrine, immune, and metabolic pathways involved in microbiota–gut–brain communication and discusses how their disruption may affect neurotransmitter signaling, inflammation, barrier function, and host metabolism. We also review current intervention strategies, including microbiota modulation, dietary and metabolic approaches, neural and immunomodulatory interventions, and behavioral and traditional Chinese medicine (TCM) approaches. Particular attention is given to the strength of the available evidence, its clinical relevance, and the limitations and safety considerations that need to be addressed before gut–brain-axis-based strategies can be more widely applied.

## 1 Introduction

The gut–brain axis describes bidirectional communication between the gastrointestinal tract and the CNS through neural, endocrine, and immune pathways. The concept has developed from early observations of the effects of psychological states on gastrointestinal function to studies of the vagus nerve and hypothalamic–pituitary–adrenal (HPA) axis as major components of brain–gut communication (Lewandowska-Pietruszka et al., 2022). More recently, advances in microbiome research have added the gut microbiota to this framework, with microbial communities and their metabolites increasingly recognized as modulators of gut–brain signaling (Cryan and Dinan, 2012; Carabotti et al., 2015).

Gut–brain communication is relevant to both gastrointestinal and neurological disorders. Altered signaling has been extensively studied in functional gastrointestinal disorders, while preclinical studies have also implicated the gut microbiota in neurodegenerative disease mechanisms, including those relevant to Parkinson’s disease (PD) (Sampson et al., 2016). Communication occurs in both directions: stress and autonomic activity can alter gastrointestinal function and the intestinal environment, whereas gut-derived signals can influence the nervous system through neural, endocrine, immune, and metabolic routes (Carabotti et al., 2015; van Thiel et al., 2020; Bautista et al., 2025; Jabbari Shiadeh et al., 2025). The relative contribution of these pathways, however, varies across experimental models and human populations.

An important limitation of the current literature is that association does not necessarily indicate causation (Wade and Hall, 2020). Differences in the gut microbiome may contribute to disease processes, but they can also reflect host and environmental factors, including differences in intestinal transit (Vandeputte et al., 2016). Findings from animal models therefore need to be distinguished from evidence obtained in humans, particularly when considering therapeutic applications (Wade and Hall, 2020). This review explains the main signaling pathways of the gut-brain axis, looks at existing intervention strategies, and focuses on how strong the supporting evidence is, the limitations in practical applications, and safety issues.

## 2 Methodology

This narrative review summarizes current evidence on the mechanisms, therapeutic strategies, and clinical translation of the microbiota–gut–brain axis. Relevant literature was searched in PubMed, Web of Science, and Embase. Search terms included “gut–brain axis,” “microbiota–gut–brain axis,” “gut microbiota,” “vagus nerve,” “HPA axis,” “intestinal barrier,” “neuroinflammation,” “short-chain fatty acids (SCFAs),” “tryptophan,” “bile acids,” “probiotics,” “prebiotics,” “synbiotics,” “fecal microbiota transplantation (FMT),” “Mediterranean diet (Med Diet),” “Low fermentable oligosaccharides, disaccharides, monosaccharides, and polyols (FODMAP) diet,” “ketogenic diet (KD),” “cognitive behavioral therapy (CBT),” “acupuncture,” “Chinese herbal medicine,” and “biomarkers.” These terms were used alone or in combination as appropriate. We also checked the reference lists of relevant reviews and key articles for additional studies.

Titles and abstracts were screened for relevance, with full texts reviewed when necessary. We considered experimental and clinical studies, randomized controlled trials, observational studies, systematic reviews, meta-analyses, consensus statements, and clinical guidelines addressing gut–brain axis mechanisms, interventions, safety, or clinical applications. Conference abstracts, articles lacking sufficient information for evaluation, studies outside the scope of gut–brain interactions, and non-English publications were excluded. Supplementary Tables S1, S2 provide an overview of the evidence levels for the studies included in this review.

## 3 Core communication mechanisms of the gut-brain axis

### 3.1 Neural pathway

Neural signaling is one of the main routes through which the gut and brain communicate in both directions (Carabotti et al., 2015; Gershon and Margolis, 2021). It involves the CNS, the enteric nervous system (ENS), and autonomic and sensory pathways that connect the gastrointestinal tract with the brain. Together, these neural networks help regulate gastrointestinal function and convey gut-derived information to the CNS, where it can influence central neural activity, as shown in Figure 1.

**Figure 1:**
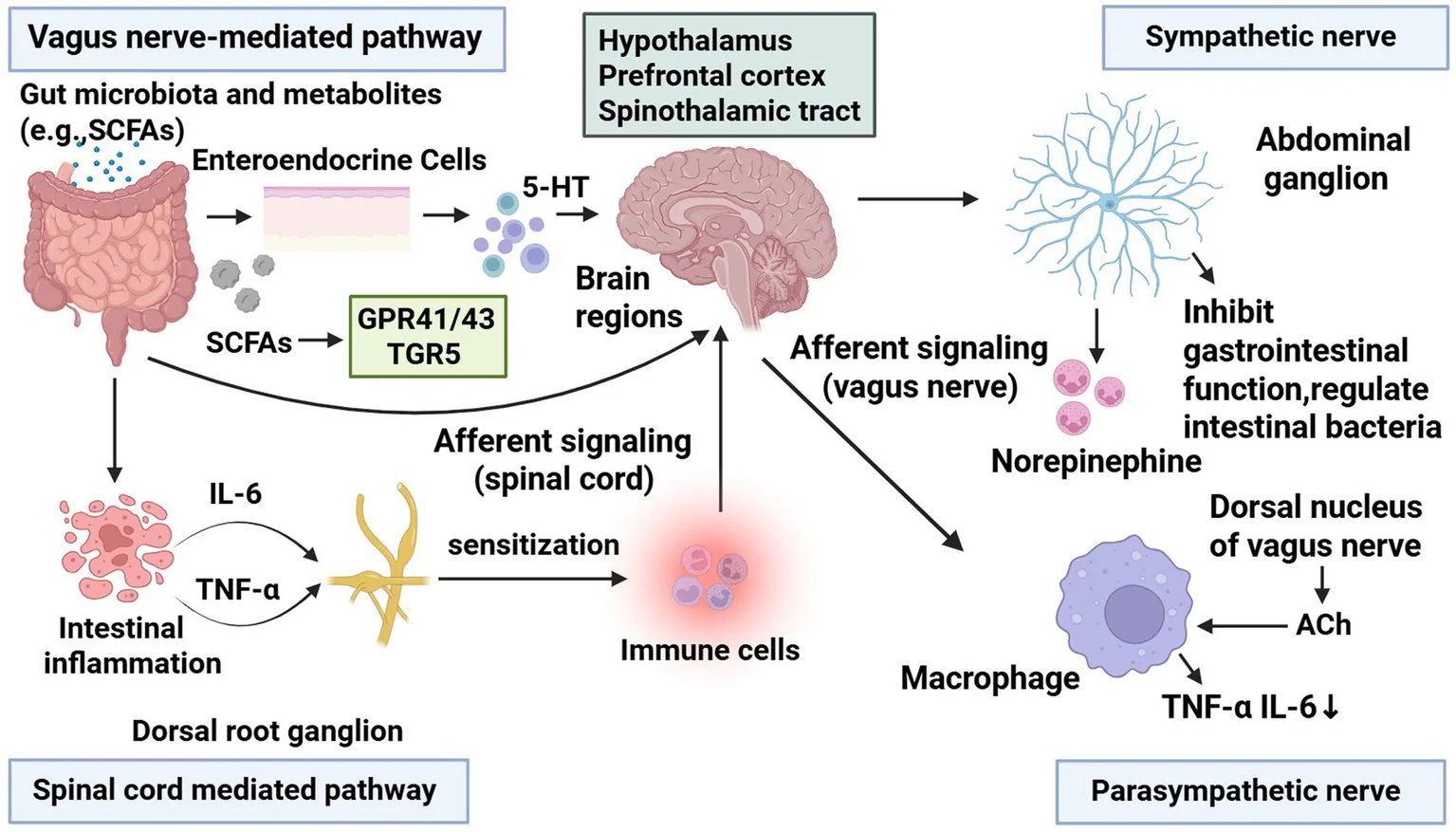
Neural pathways of the gut–brain axis. Bidirectional neural regulatory system of the gut-brain axis, including ascending and descending pathways. Ascending pathways transmit gut microbiota metabolic signals to the central nervous system via the vagus nerve to regulate metabolism and emotion, and convey intestinal nociceptive signals via the spinal cord-mediated pathway to mediate pain sensation. Descending pathways inhibit gastrointestinal function and modulate microbiota through the sympathetic nerve, whereas the parasympathetic nerve maintains intestinal immune and functional homeostasis via the cholinergic anti-inflammatory pathway. The two pathways coordinately achieve bidirectional signal transduction between the gut and brain.

The ascending pathway conveys intestinal signals to the CNS primarily through vagal and spinal afferent pathways (Carabotti et al., 2015; Gershon and Margolis, 2021). Gut microorganisms metabolize dietary components, including fiber and tryptophan, generating SCFAs and tryptophan-derived metabolites that can influence intestinal and enteroendocrine signaling (Ye et al., 2021; Koh et al., 2016). Microbial metabolites and luminal signals can influence enteroendocrine and enterochromaffin-cell signaling, including pathways involved in 5-hydroxytryptamine (5-HT) release (Ye et al., 2021; Koh et al., 2016; Reigstad et al., 2015). Released 5-HT can activate 5-HT₃ receptors on vagal afferent fibers, converting local chemical signals into neural activity (Browning, 2015). These afferent signals are relayed to the nucleus tractus solitarius (NTS) in the brainstem and integrated with neural circuits involved in autonomic, metabolic, stress-related and emotional regulation (Gershon and Margolis, 2021). Spinal afferent pathways also transmit nociceptive information arising from intestinal inflammation and mechanical or chemical stimuli. Pro-inflammatory mediators, including interleukin-6 (IL-6) and tumor necrosis factor-α (TNF-α), can sensitize visceral sensory pathways and contribute to abdominal pain and visceral hypersensitivity in gastrointestinal disorders such as irritable bowel syndrome (IBS) and inflammatory bowel disease (IBD) (Sun et al., 2025; Craig, 2002).

Descending signals from the CNS regulate intestinal function through the sympathetic and parasympathetic branches of the autonomic nervous system (Browning and Travagli, 2014). Under stress, sympathetic activation can modify gastrointestinal motility and secretion (Browning and Travagli, 2014), while norepinephrine (NE) may also affect the growth of certain intestinal bacteria (Freestone et al., 1999). Parasympathetic input, particularly through the vagus nerve, contributes to the control of intestinal inflammation. Acetylcholine (ACh) released through this pathway acts on α7 nicotinic acetylcholine receptors (α7nAChR) expressed on macrophages and suppresses pro-inflammatory signaling (Wang et al., 2003).

### 3.2 Endocrine pathway

Endocrine signaling provides another route for bidirectional communication between the gut and the brain and involves the HPA axis, enteroendocrine hormones, and microbiota-derived metabolites (Rastelli et al., 2019; Christiansen et al., 2018; Legan et al., 2022). Gut microbial metabolism can influence endocrine signaling through metabolites such as SCFAs, which can stimulate enteroendocrine cells to secrete glucagon-like peptide-1 (GLP-1) and peptide YY (PYY) (Rastelli et al., 2019; Christiansen et al., 2018). The gut microbiota can also influence host serotonin (5-HT) systems through effects on tryptophan metabolism and enterochromaffin-cell signaling (Legan et al., 2022).

Descending endocrine regulation is prominently mediated by the HPA axis (Tsigos and Chrousos, 2002). In response to stress, corticotropin-releasing hormone (CRH) released from the hypothalamus stimulates pituitary secretion of adrenocorticotropic hormone (ACTH), which in turn promotes glucocorticoid release from the adrenal cortex (Tsigos and Chrousos, 2002). The relationship between the gut microbiota and HPA-axis activity is bidirectional. Preclinical studies have shown that microbial colonization can influence HPA-axis development and stress responsiveness (Sudo et al., 2004), whereas clinical observations in hypercortisolemic conditions suggest associations among sustained glucocorticoid exposure, impaired intestinal barrier function, gut dysbiosis, and systemic inflammation (Lin et al., 2024). However, the extent to which these interactions contribute to specific human disorders remains unclear.

Ascending endocrine signaling involves hormones released by intestinal enteroendocrine cells in response to nutrients and other luminal signals (Gribble and Reimann, 2016). These hormones can influence brain function through neural and circulating routes. Cholecystokinin (CCK), for example, contributes to satiation partly by activating receptors on vagal afferent pathways that relay gastrointestinal signals to the brainstem (Moran, 2000; Owyang and Heldsinger, 2011). GLP-1 also participates in gut–brain regulation of appetite and energy balance through peripheral and central GLP-1 receptor signaling (Gribble and Reimann, 2016; Secher et al., 2014; Hölscher, 2010). PYY, particularly PYY3–36, contributes to appetite regulation and can reduce food intake through Y2 receptor-dependent mechanisms (Batterham et al., 2003). The main endocrine routes involved in gut–brain communication are summarized in Figure 2.

**Figure 2:**
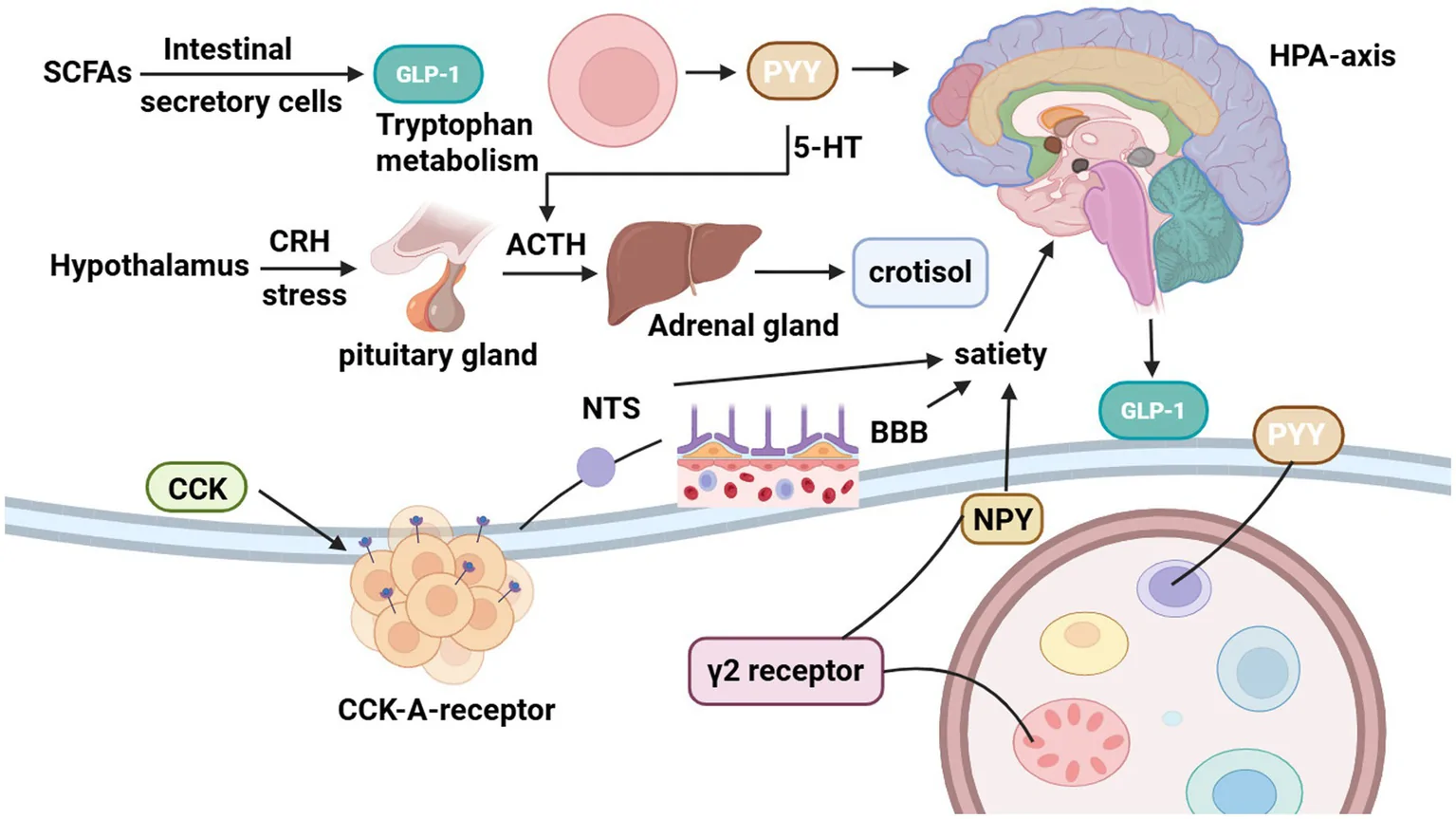
Endocrine Pathway of the gut–brain axis. Bidirectional gut-brain endocrine regulatory network centered on the HPA axis, including descending regulation, ascending feedback, and microbial auxiliary regulation. Descending signals from the HPA axis trigger cortisol secretion to modulate intestinal function and barrier integrity. Ascending signals are mediated by enteroendocrine cells that secrete CCK, GLP-1, PYY and other hormones to induce satiety and suppress appetite. Gut microbial metabolism generates SCFAs and regulates 5-HT synthesis, which assist in completing gut-brain endocrine signal crosstalk.

### 3.3 Immune pathway

Immune signaling represents an important component of microbiota–gut–brain communication and is closely linked to microbial metabolism. SCFAs, tryptophan-derived metabolites and bile acids can interact with intestinal epithelial and immune cells, thereby influencing barrier integrity, innate immune signaling and cytokine production. Under physiological conditions, microbial metabolites can contribute to intestinal immune homeostasis. For example, the SCFA butyrate promotes the differentiation of colonic regulatory T (Treg) cells in experimental models (Furusawa et al., 2013). SCFAs and tryptophan-derived indole metabolites can also support epithelial barrier function and modulate inflammatory signaling, while bile acids participate in immune regulation through receptors such as farnesoid X receptor (FXR) and takeda G protein-coupled receptor 5 (TGR5). The direction and magnitude of these effects depend on the specific metabolite, receptor pathway and host context (Fu et al., 2023; Di Ciaula et al., 2023).

Dysbiosis can alter microbial metabolic output, including the production of SCFAs and tryptophan-derived metabolites, and can modify the composition of the intestinal bile acid pool. Together with impaired barrier integrity, these changes may increase mucosal exposure to microbial products and tissue-derived danger signals, promoting innate immune activation. Recognition of these signals by pattern-recognition receptors can activate inflammatory pathways, including toll like receptors (TLR) and inflammasome-associated signaling, and increase the production of pro-inflammatory mediators such as interleukin-1β (IL-1β), IL-6 and TNF-α (Fu et al., 2023; Dantzer, 2018; Elinav et al., 2011; Buhner and Schemann, 2012; Henao-Mejia et al., 2012). Reduced SCFA and indole-associated anti-inflammatory signaling and dysregulated bile acid signaling may further modify these responses, although their effects are not uniform across metabolites or disease settings (Fu et al., 2023; Di Ciaula et al., 2023).

Intestine-derived immune signals may influence the CNS through circulating mediators and neural pathways. Circulating inflammatory mediators can act on brain vascular endothelial cells and alter blood–brain barrier (BBB) function, thereby contributing to neuroimmune activation (Henao-Mejia et al., 2012; Galea, 2021). Microbial metabolites may also influence BBB function. In particular, experimental evidence suggests that SCFAs can support BBB integrity, whereas altered bile acid signaling has been linked to BBB and neuroimmune function (Fock and Parnova, 2023; Xing et al., 2023). Systemic inflammation can also increase immune-cell trafficking across the BBB, but the extent and consequences of this process vary with the inflammatory state and disease context (Galea, 2021; Huang et al., 2021). In parallel, vagal afferent pathways provide a neural route through which intestinal signals can reach the brainstem, with the NTS serving as a major relay for visceral sensory information (Ye et al., 2021; Powley, 2021). Within the CNS, activated microglia can release inflammatory and oxidative mediators that affect neuronal signaling and synaptic function (Hoogland et al., 2015; Zhang et al., 2017; Zhu et al., 2010). Persistent neuroimmune activation has been associated with neurobehavioral and cognitive disturbances, although these outcomes are disease- and context-dependent (Hoogland et al., 2015; Zhang et al., 2017; Zhu et al., 2010). Intestinal immune and metabolic signals may therefore influence the CNS through several routes, although the contribution of each pathway is likely to vary across diseases and experimental settings.

The brain can in turn influence intestinal immune function, barrier integrity and microbial composition through autonomic and endocrine pathways (Sudo et al., 2004; Ouyang et al., 2025; Tay et al., 2025; Scott et al., 2018). Activation of the HPA axis increases glucocorticoid secretion, while sympathetic signaling alters intestinal NE availability (Sudo et al., 2004; Ouyang et al., 2025; Tay et al., 2025; Scott et al., 2018). These mediators have context-dependent effects on intestinal immunity. Glucocorticoids can suppress acute inflammatory responses, whereas prolonged exposure may impair epithelial barrier function and alter macrophage and T-cell activity (Luo et al., 2023; Khadka et al., 2024). NE can also modify immune signaling and influence the growth or behavior of some intestinal bacteria (Sharma and Casey, 2014). In parallel, vagal efferent signaling can limit inflammatory responses through the cholinergic anti-inflammatory pathway, in which ACh acts on α7nAChR expressed by immune cells and reduces pro-inflammatory signaling (Wang et al., 2003; Borovikova et al., 2000). These neural and endocrine pathways therefore provide routes through which central physiological states can modify intestinal immune and microbial environments.

### 3.4 Microbial metabolic pathway

Gut microorganisms generate a wide range of metabolites that can influence intestinal and host physiology, including SCFAs, tryptophan-derived metabolites and secondary bile acids (Keitel et al., 2010; Agus et al., 2021). Their biological effects depend on metabolite availability, receptor distribution, and the route by which signals reach host tissues. These effects may be mediated locally or through epithelial, enteroendocrine, immune, or neural pathways. Importantly, the detection of a neuroactive compound in the intestinal lumen does not imply direct access to the CNS. Peripheral serotonin (5-HT), for example, does not readily cross the blood–brain barrier and therefore does not replenish central 5-HT pools (Jones et al., 2020). Accordingly, microbial production of a neuroactive compound alone should not be interpreted as evidence of direct brain exposure or therapeutic activity. The major microbial metabolic pathways involved in gut–brain communication are summarized in Figure 3.

**Figure 3:**
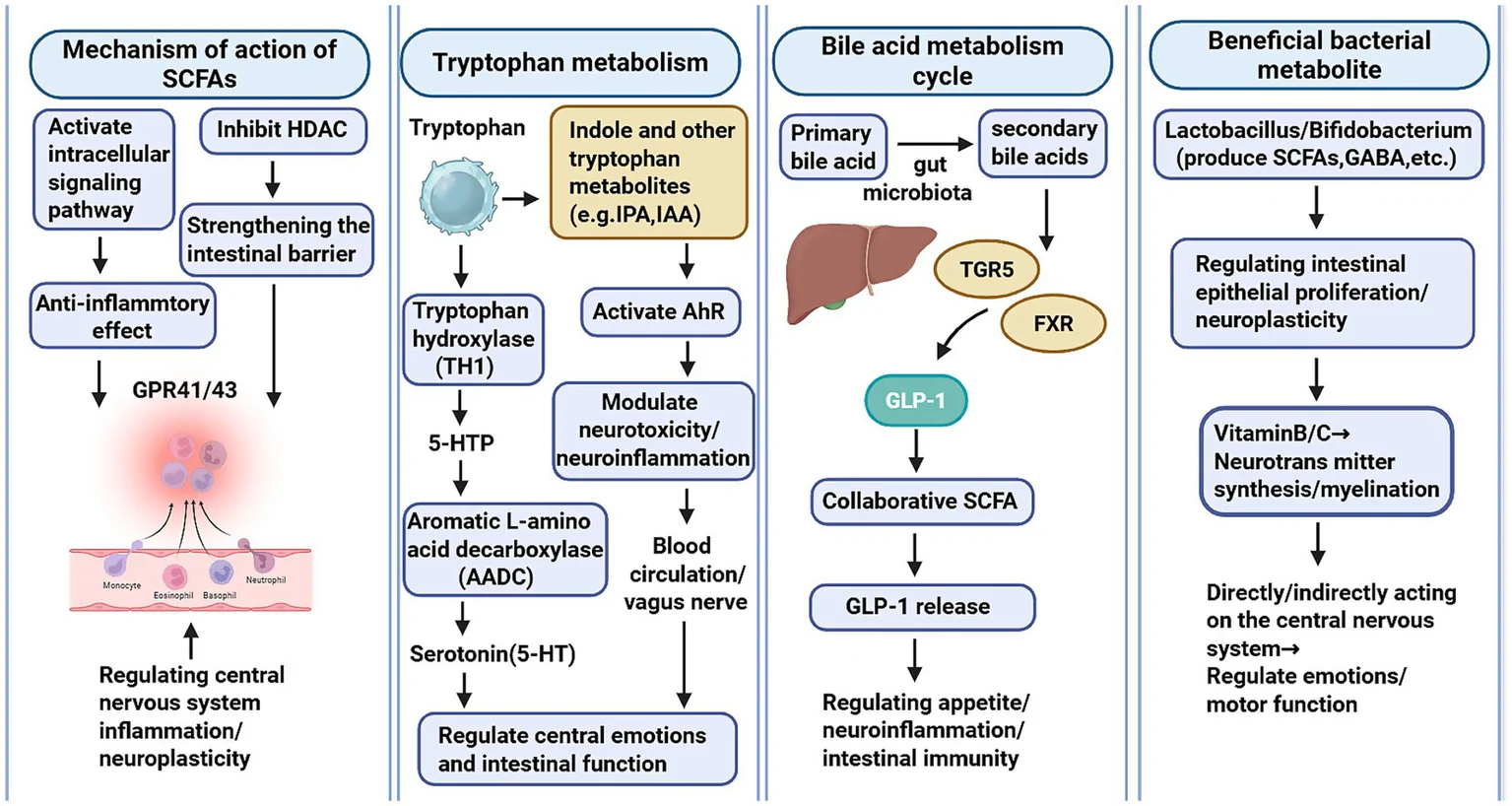
Microbial Metabolic Pathway of the gut–brain axis. Gut microbiota-derived metabolites, including SCFAs, tryptophan-derived metabolites, secondary bile acids, and other bioactive compounds, may influence gut–brain communication through epithelial, enteroendocrine, immune, and neural pathways. The effects of these metabolites depend on their biological context and may involve both local and systemic signaling.

SCFAs are major products of microbial fermentation of dietary substrates and can influence intestinal epithelial, immune and enteroendocrine functions. They signal through receptors including GPR41/FFAR3 and GPR43/FFAR2 and can also regulate gene expression through inhibition of histone deacetylases (Maslowski et al., 2009; Li et al., 2018; Nøhr et al., 2015; Davie, 2003). These mechanisms have been associated with modulation of inflammatory responses, epithelial barrier function and enteroendocrine signaling, although much of the mechanistic evidence is derived from experimental models. SCFAs may also influence the gut–brain axis indirectly through immune, endocrine and neural pathways. Experimental evidence further suggests that circulating SCFAs can influence blood–brain barrier and neuroimmune function, but these effects should not be interpreted solely in terms of direct brain penetration (Fock and Parnova, 2023).

Tryptophan metabolism represents another route through which the gut microbiota can influence intestinal and host physiology. Gut bacteria convert dietary tryptophan into a range of indole derivatives, including indole-3-propionic acid (I3PA) (Zünd et al., 2025). Some microbial indole metabolites can act as signaling molecules through host receptors such as the aryl hydrocarbon receptor (AhR), although their receptor activity and downstream effects vary among individual metabolites and biological contexts (Lamas et al., 2016; Miyamoto et al., 2024). By contrast, serotonin biosynthesis in intestinal enterochromaffin cells is mediated by host enzymes. Tryptophan hydroxylase 1 (TPH1) converts tryptophan to 5-hydroxytryptophan (5-HTP), which is subsequently converted to peripheral serotonin (5-HT) by aromatic L-amino acid decarboxylase (Yano et al., 2015). Gut microorganisms and their metabolites can influence host serotonin production (Furusawa et al., 2013; Yano et al., 2015), but current evidence does not support presenting IPA–AhR signaling as a direct pathway driving 5-HT biosynthesis. Tryptophan-derived indole metabolites have also been implicated in the regulation of intestinal barrier and immune function, including AhR-dependent signaling (Lamas et al., 2016; Miyamoto et al., 2024). In parallel, the kynurenine pathway is predominantly mediated by host enzymes, including indoleamine 2,3-dioxygenase (IDO) in peripheral tissues and tryptophan 2,3-dioxygenase (TDO), which is expressed mainly in the liver. Gut microbiota can nevertheless influence host tryptophan availability and immune signaling and may thereby alter the balance between indole, serotonin and kynurenine metabolism (Miyamoto et al., 2024). These interactions provide a potential link between microbial tryptophan metabolism and host immune and neuroactive signaling, although their relative contributions and causal relevance vary across experimental and disease contexts.

Bile acid metabolism provides another link between the gut microbiota and host signaling. Intestinal microorganisms convert primary bile acids synthesized by the liver into a diverse pool of secondary bile acids (Ridlon et al., 2006). Bile acids can signal through host receptors, including FXR and TGR5, and thereby influence metabolic, enteroendocrine and immune functions (Ridlon et al., 2006; Thomas et al., 2009; Tolhurst et al., 2012). For example, microbiota-dependent bile acid signaling through intestinal TGR5 has been implicated in the regulation of GLP-1 secretion (Wang et al., 2023). Changes in the composition of the bile acid pool during dysbiosis may affect epithelial barrier and inflammatory signaling. However, the effects of secondary bile acids are not uniformly protective or detrimental and depend on the specific bile acid species, concentration, receptor pathway and disease context (Di Ciaula et al., 2023). Altered bile acid metabolism has also been linked to neuroimmune and blood–brain barrier function, although its causal contribution to neurological disease remains incompletely established (Xing et al., 2023).

Other microbial metabolites and neuroactive compounds may also contribute to gut–brain communication. Certain gut microorganisms are capable of producing γ-aminobutyric acid (GABA), and microbial glutamate decarboxylase catalyzes the conversion of glutamate to GABA (Strandwitz, 2018; Belelli et al., 2025). This reaction should not be confused with the biosynthesis of dopamine or its precursors. In mice, specific *Lactobacillus* strains have been reported to alter GABA receptor expression in the brain and behavioral responses in a vagus-dependent manner (Bravo et al., 2011), although these findings are strain- and model-specific. Microbial polyamines may also influence intestinal barrier and host metabolic functions, but their contribution to gut–brain signaling in humans remains less well defined (Zhu et al., 2021). More broadly, SCFAs, tryptophan-derived metabolites, bile acids and other microbial products interact with host immune, endocrine and neural pathways rather than functioning as isolated mechanisms. Gut microbiota also influences the intestinal luminal metabolome (Matsumoto et al., 2012), while neural and endocrine signals from the host can in turn modify the intestinal environment. These findings support bidirectional communication within the microbiota–gut–brain axis, although the relative contribution and clinical relevance of individual metabolic pathways remain context-dependent.

## 4 Intervention strategies targeting the gut–brain axis

### 4.1 Microbiota modulation

#### 4.1.1 Probiotics

Probiotics are live microorganisms that, when administered in adequate amounts, confer a health benefit on the host (Hill et al., 2014). Their effects are strain- and context-specific and may involve interactions with resident microbiota, microbial metabolites, epithelial barrier function and host immune signaling (Hill et al., 2014; Sánchez de Medina et al., 2013; Yadav et al., 2017; Macfarlane and Macfarlane, 2003; Mann et al., 2024). These mechanisms are not shared uniformly across probiotic strains or clinical populations.

Preclinical studies have examined whether probiotics can modify microbiota–gut–brain pathways in neurological disease. In animal models of Alzheimer’s disease (AD), probiotic administration has been associated with changes in gut microbial composition, inflammatory signaling, barrier-related pathways and cognitive performance (Siripaopradit et al., 2024). Some studies have also reported changes in amyloid-β (Aβ) deposition, neuroinflammation and neurotrophic signaling (Siripaopradit et al., 2024). Human trials in AD and mild cognitive impairment have produced mixed results, with meta-analyses suggesting possible cognitive benefits but substantial heterogeneity and limited study numbers (Zhu et al., 2021).

Human evidence for psychological outcomes is also heterogeneous. In patients with IBS, probiotic treatment has been associated with changes in microbial and metabolic profiles together with improvement in psychological comorbidities (Martin et al., 2024). Studies in healthy or stressed populations have reported psychological or gastrointestinal effects following probiotic supplementation (Tran et al., 2019; Kato-Kataoka et al., 2016), but these findings should not be interpreted as treatment evidence for major depressive disorder (MDD). In patients with MDD, systematic reviews suggest that probiotics may reduce depressive symptoms in some studies (Lin et al., 2023), while changes in gut microbial composition have not been consistent across trials (Ng et al., 2023).

Probiotic effects depend on the strain, formulation, dose and target population, and results from one product or clinical setting cannot be generalized to others. Current evidence does not support probiotics as established treatments for AD or MDD, and rare invasive infections have been reported in vulnerable patients (Costa et al., 2018). Probiotics are therefore better considered investigational adjunctive approaches in these conditions.

#### 4.1.2 Prebiotics

Prebiotics are substrates that are selectively utilized by host microorganisms and confer a health benefit (Burokas et al., 2017)^.^ Their effects vary with the type and dose of substrate, host characteristics and baseline microbiota, making it difficult to generalize findings across populations. In mice exposed to chronic psychosocial stress, supplementation with fructo-oligosaccharides and galacto-oligosaccharides attenuated stress-related changes in gut microbial composition and neuroendocrine responses and reduced anxiety- and depression-related behaviors (Burokas et al., 2017). These findings support a role for prebiotics in microbiota–gut–brain signaling in experimental models, but comparable effects in patients with neuropsychiatric disorders remain less well established.

Inulin-type fructans (ITF) are among the most extensively studied prebiotic fibers. In overweight and obese men, inulin fermentation increased SCFA production and was accompanied by increased fat oxidation (van der Beek et al., 2018). In the Food4Gut randomized placebo-controlled trial in patients with obesity, ITF supplementation altered *Bifidobacterium* abundance and fecal SCFA profiles and improved fecal markers related to intestinal inflammation (Neyrinck et al., 2021). These findings indicate that ITF can influence microbial and metabolic features in humans, although the clinical significance of these changes and their relevance to long-term weight management remain uncertain.

Prebiotic responses vary with substrate, dose and population, and human evidence for metabolic and neuropsychiatric disorders remains heterogeneous. Higher intakes can also cause gastrointestinal symptoms such as bloating and flatulence (Gibson et al., 2017). Prebiotics should therefore not be considered established treatments for obesity or neuropsychiatric disorders on the basis of current evidence.

#### 4.1.3 Synbiotics: synergistic action of probiotics and prebiotics

A synbiotic is defined as a mixture comprising live microorganisms and substrate(s) selectively utilized by host microorganisms that confers a health benefit on the host (Swanson et al., 2020). They can be classified as complementary or synergistic synbiotics according to the relationship between the microbial and substrate components. In complementary synbiotics, established probiotic and prebiotic components are combined but may act independently, whereas in synergistic synbiotics the substrate is specifically intended to support the co-administered microorganism(s) (Swanson et al., 2020). Depending on their composition, synbiotic formulations may influence resident microbial communities, microbial metabolism, and intestinal barrier and immune functions (Swanson et al., 2020; van Zanten et al., 2014). However, these effects depend on the specific formulation and host context, and not all combinations necessarily result in synergistic effects.

PD is frequently accompanied by gastrointestinal dysfunction, and constipation is one of its most common non-motor manifestations (Fasano et al., 2015; Adams-Carr et al., 2016). In a randomized, double-blind, placebo-controlled trial involving 120 patients with PD and constipation, a fermented milk containing multiple probiotic strains and prebiotic fiber improved constipation-related outcomes compared with placebo (Barichella et al., 2016). Because this intervention contained both probiotic and prebiotic components, it may be considered relevant to combined microbiota-directed strategies. However, the trial primarily evaluated gastrointestinal rather than neurological outcomes. A separate randomized trial of multistrain probiotics without a prebiotic component also reported improvement in constipation-related outcomes (Tan et al., 2021).

Clinical evidence for microbiota-directed interventions in PD remains limited, particularly for synbiotic formulations. Trials of probiotic-based interventions have generally involved relatively small samples and short intervention periods and have focused mainly on constipation and other gastrointestinal outcomes, while evidence for neurological benefits and long-term efficacy remains limited (Yin and Zhu, 2022). Mild gastrointestinal adverse events, including abdominal pain and bloating, have been reported, although serious treatment-related adverse events appear uncommon in the available trials (Yin and Zhu, 2022). Larger randomized trials with longer follow-up and well-defined formulations are needed to determine whether synbiotics provide benefits beyond the management of gastrointestinal symptoms in PD.

#### 4.1.4 FMT

FMT involves the transfer of processed stool from a screened donor to a recipient with the aim of modifying the recipient gut microbial community. Its best-established clinical application is the prevention of recurrence in selected patients with recurrent *Clostridioides difficile* infection (rCDI), whereas its use in metabolic, neurological and psychiatric disorders remains investigational (Yadegar et al., 2024).

Beyond rCDI, several small clinical studies have examined the metabolic effects of FMT. Transfer of microbiota from lean donors has been associated with improved insulin sensitivity in individuals with metabolic syndrome (Vrieze et al., 2012), while subsequent trials have investigated FMT, alone or combined with fiber supplementation, in obesity and metabolic syndrome (Mocanu et al., 2021). However, clinical responses have been variable, and changes in microbial composition or engraftment should not by themselves be interpreted as evidence that microbiota alterations mediate the observed metabolic effects (Vrieze et al., 2012; Mocanu et al., 2021; Zecheng et al., 2023).

Preclinical studies provide stronger evidence that gut microbial communities can influence neurobehavioral phenotypes. Transfer of fecal microbiota from patients with MDD to microbiota-depleted or germ-free rodents has induced depression-like behavioral and metabolic changes (Kelly et al., 2016; Zheng et al., 2016). These experiments support a causal contribution of transferred microbial communities to behavioral phenotypes in animal models, but they do not establish that microbiota dysbiosis is a primary cause of MDD in humans. Changes in tryptophan metabolism, immune signaling and other microbiota–host interactions have been proposed as possible links between the gut microbiota and these behavioral effects (Cryan and Dinan, 2012; Kelly et al., 2016; Zheng et al., 2016; Erny et al., 2015), but their contribution in patients remains uncertain.

Outside its established use in selected patients with rCDI, FMT remains experimental for metabolic and neuropsychiatric disorders. Variable microbial engraftment and the risk of transmitting pathogens, including antimicrobial-resistant organisms, remain important concerns (Ianiro et al., 2022; Goldsmith et al., 2024). Differences in donor screening, preparation protocols and regulatory frameworks further limit standardization and routine use in non-rCDI indications (Yadegar et al., 2024; Goldsmith et al., 2024).

### 4.2 Metabolic substrate modulation

#### 4.2.1 Med diet

The Med Diet is characterized by a high intake of plant-based foods, moderate consumption of high-quality protein, and a predominance of unsaturated fats. Higher adherence to this dietary pattern has been associated with differences in gut microbial composition and metabolomic profiles, while a 1-year Med Diet intervention in older adults induced microbiome changes across five European countries (De Filippis et al., 2016; Ghosh et al., 2020). These effects may be related, at least in part, to the range of substrates available for microbial metabolism.

Dietary fiber and resistant starch from whole grains, legumes, fruits, and vegetables provide fermentable substrates for gut bacteria and support microbial production of SCFAs (De Filippis et al., 2016; Ghosh et al., 2020). Polyphenol-rich foods, including fruits, vegetables, nuts, and extra-virgin olive oil, may also influence microbial composition and metabolic activity (Fraga et al., 2019). Accordingly, greater adherence to the Med Diet has been associated with differences in SCFA-producing taxa and microbial metabolites (De Filippis et al., 2016; Ghosh et al., 2020). SCFAs, particularly butyrate, may contribute to intestinal and systemic anti-inflammatory effects through regulation of inflammatory signaling pathways, including nuclear factor kappa-B (NF-κB) signaling (Segain et al., 2000). The magnitude and direction of these microbial and metabolic changes, however, are not uniform across individuals.

The effects of the Med Diet are not confined to the gut. In a randomized clinical trial, a Med Diet supplemented with extra-virgin olive oil or nuts was associated with better cognitive performance than a control diet among older adults at high cardiovascular risk (Valls-Pedret et al., 2015). An observational study also found that greater Med Diet adherence was associated with more favorable Alzheimer’s disease biomarker profiles and less brain atrophy (Ballarini et al., 2021). Evidence for individual dietary components is also emerging. In a randomized controlled trial involving individuals with mild cognitive impairment, extra-virgin olive oil supplementation was associated with changes in BBB permeability and functional brain connectivity (Kaddoumi et al., 2022).

These findings do not, however, establish that changes in the gut microbiota are responsible for the cognitive effects associated with the Med Diet. The diet can also influence metabolic, inflammatory, and vascular processes, and much of the literature linking Med Diet adherence with cognitive outcomes is observational (Petersson and Philippou, 2016). In the 1-year intervention reported by Ghosh et al., diet-responsive microbial taxa were associated with cognitive and inflammatory measures, but this does not demonstrate that the microbial changes mediated the cognitive effects (Ghosh et al., 2020). Further longitudinal and intervention studies incorporating repeated microbiome measurements alongside cognitive and metabolic outcomes will be needed to clarify this relationship.

#### 4.2.2 Low FODMAP diet

The low FODMAP diet is a structured dietary intervention for selected patients with IBS, rather than a long-term elimination diet. Restriction of fermentable oligosaccharides, disaccharides, monosaccharides and polyols reduces exposure to poorly absorbed and rapidly fermentable carbohydrates, thereby limiting luminal distension related to osmotic effects and fermentation in susceptible patients (van Lanen et al., 2021; Halmos et al., 2014). Randomized trials and meta-analyses have shown improvements in global IBS symptoms, abdominal pain and bloating during the restriction phase (van Lanen et al., 2021; Halmos et al., 2014; Schumann et al., 2018; Black et al., 2022).

Clinical implementation generally involves three stages: short-term restriction, systematic reintroduction and subsequent personalization according to individual symptom tolerance (McKenzie et al., 2016; Lomer, 2024). This distinction is important because prolonged strict restriction is not intended as the final form of the diet. Low FODMAP intake can alter the colonic microbial environment, and concerns remain regarding nutritional adequacy and unfavorable microbial changes when restriction is maintained unnecessarily (Lomer, 2024; O'Keeffe et al., 2018). Reintroduction allows tolerated FODMAP-containing foods to be restored while identifying individual dietary triggers.

The low FODMAP diet is supported for symptom management in selected patients with IBS, but it should not be generalized as a treatment for neurological, psychiatric or metabolic disorders. Individual responses vary, and much of the trial evidence concerns the initial restriction phase rather than long-term personalized use (Black et al., 2022). Dietitian-guided reintroduction and personalization are preferable where available, particularly when prolonged dietary restriction raises concerns about nutritional adequacy (McKenzie et al., 2016; Lomer, 2024).

#### 4.2.3 KD

The KD is a high-fat, very-low-carbohydrate dietary therapy with an established role in selected patients with drug-resistant epilepsy, particularly in pediatric practice (Lindefeldt et al., 2019; Desli et al., 2022). Carbohydrate restriction promotes hepatic ketogenesis and shifts energy metabolism toward greater use of ketone bodies. The antiseizure effects of the KD are unlikely to depend on a single mechanism and have been linked to changes in cellular energetics, neurotransmission, ion-channel activity and inflammatory signaling (Masino and Rho, 2012).

The KD can also alter gut microbial composition. In children with severe epilepsy, ketogenic therapy produced changes in both the taxonomic and functional composition of the gut microbiota (Lindefeldt et al., 2019). Preclinical studies further suggest that the microbiota may contribute to seizure protection; in mouse models, microbiota depletion impaired the antiseizure effect of the KD, whereas selected KD-associated bacteria restored protection (Olson et al., 2018). However, these findings do not establish that microbiota modulation is the primary mechanism responsible for the clinical efficacy of the KD in humans. Microbiota-related effects should therefore be considered alongside, rather than as a replacement for, the metabolic and neuronal mechanisms proposed for the KD (Boison, 2017).

A detailed comparison of the three strategies in this section is summarized in Supplementary Table S2.

The KD therapy can be effective in drug-resistant epilepsy, but adherence is demanding and gastrointestinal, metabolic and nutritional adverse effects require monitoring (Desli et al., 2022; Kossoff et al., 2018). The established indication discussed here is drug-resistant epilepsy (Kossoff et al., 2018), and evidence from this population should not be extrapolated to obesity, psychiatric disorders or other neurological conditions.

### 4.3 Neural / immunomodulators

Escitalopram (ESC), a widely used selective serotonin reuptake inhibitor (SSRI), may influence peripheral metabolism and the gut microbiota in addition to its established central pharmacological actions. In a longitudinal multi-omics study of patients with MDD, 12 weeks of ESC treatment partially normalized altered plasma metabolic profiles, with increases in several amino acids that were depleted in MDD and decreases in fatty acids that were elevated before treatment (Wang et al., 2023). ESC also exerted a modest inhibitory effect on the gut microbiota, whereas patients who achieved remission had greater baseline microbial richness and a more resilient microbial community than non-remitters (Wang et al., 2023). Gut microbial features were associated with changes in circulating metabolites, including tryptophan and the microbiota-derived metabolite I3PA (Wang et al., 2023). ESC treatment was accompanied by changes in gut microbial features and peripheral metabolism that were also related to clinical response. However, these findings do not show that the antidepressant effect of ESC is mediated by the gut microbiota. It also remains unclear how much of the observed metabolic change reflects direct drug action and how much is linked to microbiota-related processes.

In mouse models fed a high-fat diet, semaglutide altered the gut microbiota and partially reversed diet-related dysbiosis. Changes were observed in several taxa, including *Alloprevotella*, *Alistipes*, *Akkermansia*, and *Romboutsia* (Duan et al., 2024; Feng et al., 2024). Evidence in humans is still limited. In a small self-controlled study of 15 patients with type 2 diabetes, 12 weeks of semaglutide treatment was associated with shifts in both gut microbial composition and metabolic profiles (Chen et al., 2025). Because the study included only a small number of participants and lacked a parallel control group, it cannot determine whether the microbial changes contributed to semaglutide’s metabolic effects or occurred as a consequence of treatment. Semaglutide also has well-established metabolic actions that do not depend on the gut microbiota. As a GLP-1 receptor agonist, it enhances glucose-dependent insulin secretion and influences appetite through both peripheral and central pathways, leading to lower energy intake and body weight (Wilding et al., 2021).

ESC and GLP-1 receptor agonists should therefore not be considered microbiota-targeted therapies. Their clinical effects are mainly explained by their known pharmacological targets, whereas the role of microbiota-related changes in treatment response is still uncertain (Wang et al., 2023; Chen et al., 2025). This is particularly relevant for semaglutide, for which current human microbiome data come from small and inadequately controlled studies (Chen et al., 2025). Better-controlled longitudinal studies that combine microbiome and metabolomic measurements will be needed to determine whether microbial changes contribute to treatment response or simply follow drug-induced metabolic changes.

### 4.4 CBT

CBT is one of the best-studied psychological interventions for IBS. By addressing maladaptive thoughts, coping behaviors, symptom-related anxiety and hypervigilance, CBT aims to modify central processes involved in the perception and interpretation of gastrointestinal symptoms. Its clinical efficacy is supported by a systematic review and network meta-analysis of 41 randomized controlled trials involving 4,072 patients, which found that both face-to-face and minimal-contact CBT reduced persistent IBS symptoms, although the overall evidence was limited by risk of bias and possible publication bias (Black et al., 2020).

Recent studies suggest that CBT response may also be accompanied by changes in the brain–gut–microbiome axis. Compared with non-responders, CBT responders showed distinct baseline gut microbial profiles and higher fecal serotonin concentrations (Jacobs et al., 2021). Following treatment, responders exhibited reduced functional connectivity in sensorimotor, brainstem, salience and default-mode networks, together with changes in white-matter microstructure (Jacobs et al., 2021; Goodoory et al., 2024). Changes in brain connectivity were also correlated with shifts in gut microbial composition, including an expansion of *Bacteroides* in responders (Jacobs et al., 2021). These findings suggest coordinated changes in brain and gut microbial features during successful CBT, but they do not establish whether microbial alterations contribute to treatment response or occur as a downstream consequence of central or behavioral changes.

Current studies are generally small, and microbiome-based predictors of CBT response still lack independent validation (Jacobs et al., 2021). It also remains unclear whether the observed microbial changes contribute to symptom improvement or occur as a consequence of treatment-related changes in brain function or behavior. Larger prospective studies are needed to clarify these relationships and assess their clinical relevance.

### 4.5 TCM

TCM including acupuncture and Chinese herbal medicine, has been investigated as an adjunctive approach for PD. In a clinical study, 8 weeks of scalp–abdominal electroacupuncture combined with conventional antiparkinsonian treatment was associated with improvements in motor function, activities of daily living and selected non-motor outcomes, together with changes in gut microbial composition, including increased *Bacteroides* and *Parasutterella* and decreased *Dialister* and *Hungatella* (Nazarova et al., 2022). However, microbiome analysis was performed only before and after treatment in the intervention group, so these findings show an association between treatment and microbial changes rather than a microbiota-mediated therapeutic mechanism.

Chinese herbal medicine is also used in PD management. A hospital-based retrospective analysis of 3,206 patient encounters documented its frequent use in real-world clinical practice (Lyu et al., 2024). Clinical evidence extends beyond observational studies: a systematic review and meta-analysis of 14 randomized double-blind placebo-controlled trials involving 1,311 patients found that herbal formulas used as adjunctive therapy were associated with improvements in several Unified Parkinson’s Disease Rating Scale domains, quality of life and non-motor symptoms. Evidence for herbal monotherapy, however, remained insufficient (Shan et al., 2018).

Evidence directly linking Chinese herbal medicine to microbiota–gut–brain modulation in PD remains largely preclinical. In 1-methyl-4-phenyl-1,2,3,6-tetrahydropyridine-treated mice, *Gastrodia elata* polysaccharide altered gut microbial composition, increased colonic SCFAs and occludin expression, and reduced neuroinflammatory markers and dopaminergic neuronal loss (Gan et al., 2024). These findings support a possible interaction among herbal interventions, gut microbiota, intestinal barrier function and neuroinflammation, but comparable mechanisms have not yet been demonstrated in patients. Future clinical studies should therefore combine standardized TCM interventions with longitudinal microbiome and metabolomic analyses, clinical outcomes and safety assessment to determine whether microbiota changes contribute to therapeutic responses.

### 4.6 Biomarkers for gut-brain-axis dysfunction

Several types of biomarkers have been investigated in the context of altered gut–brain communication. Fecal calprotectin and lactoferrin are widely used as non-invasive markers of intestinal inflammation, whereas human β-defensin 2 (HBD2) reflects mucosal innate immune activity (Dai et al., 2020). None of these markers is specific to dysfunction of the gut–brain axis, but they can still provide useful information about intestinal inflammation in disorders involving altered gut–brain communication. In patients with IBD and IBS, fecal calprotectin and HBD2 have shown value in distinguishing between the two conditions, and their performance may be improved when combined with microbiome data (Gacesa et al., 2021).

Intestinal barrier function has also been examined as a potential source of biomarkers. Zonulin is often measured as an indirect indicator of intestinal permeability, although its interpretation is problematic. Commercial assays commonly used to measure zonulin may detect proteins other than pre-haptoglobin-2 and do not provide a reliable direct measure of intestinal permeability (Massier et al., 2021). Zonulin should therefore be interpreted cautiously rather than regarded as an established stand-alone marker of impaired intestinal barrier function (Massier et al., 2021).

Microbial and metabolic measurements represent another group of candidate biomarkers. Changes in SCFAs and tryptophan-related metabolites have been reported in psychiatric disorders, particularly depression, although the direction and magnitude of these changes are not consistent across studies (Ogyu et al., 2018; Lukić et al., 2022). Microbiome profiles have likewise been investigated as potential biomarkers in psychiatric and neurological disorders, but differences in study design, population characteristics and data quality continue to limit their reproducibility (Nikolova et al., 2021). Diet, medication use, and intestinal transit can also contribute to microbiome variation (Falony et al., 2016; Vujkovic-Cvijin et al., 2020), as can differences in sample handling and analytical methods (Sinha et al., 2017).

No single biomarker has been established for assessing gut–brain-axis dysfunction across clinical disorders. Combining microbial and metabolic features has shown promise for distinguishing specific patient groups, with some models retaining good performance in validation cohorts (Yang et al., 2020). However, these models have largely been developed for individual disorders, and it is not yet clear how well they will perform across different populations or clinical settings.

## 5 Challenges and future perspectives

Research on the microbiota–gut–brain axis has expanded rapidly, but translation of these findings into clinical practice remains limited. Major obstacles include inconsistent results across microbiome studies, difficulty distinguishing association from causation, heterogeneity in intervention studies, and unresolved safety and regulatory issues. These limitations need to be considered when interpreting current evidence and when designing future studies.

### 5.1 Limitations in current gut-brain-axis research

#### 5.1.1 Methodological and interpretive limitations

Reproducibility remains a major challenge in microbiome research. Differences in sample collection, laboratory processing, sequencing platforms and bioinformatic pipelines can alter the microbial profiles obtained from the same or comparable biological material. In the Microbiome Quality Control project, analyses performed across multiple laboratories showed that biospecimen type, DNA extraction, sample handling and bioinformatic procedures all contributed to variation in taxonomic profiles (Sinha et al., 2017). Sampling niche is another source of heterogeneity: microbial profiles obtained from colonic biopsies can differ substantially from those obtained from stool (Vandeputte et al., 2016). Gastrointestinal transit also needs to be considered, because stool consistency and transit-related variation are associated with microbial richness and community composition (Nowicki et al., 2023). Medication use, diet, geography and other host characteristics introduce further variation. These methodological and biological differences make direct comparisons between cohorts difficult and may partly explain why microbial signatures reported in one population are not consistently reproduced in another.

Interpretation presents a separate problem. Most human microbiome studies are observational and therefore cannot determine whether a microbial alteration contributes to disease, results from the disease itself, or reflects a shared confounding factor. Lifestyle, medication and dietary factors can contribute to these associations, and reverse causation remains difficult to exclude (Wade and Hall, 2020). Changes in the microbiome after an intervention should therefore not automatically be interpreted as the mechanism responsible for clinical improvement. Evidence is stronger when observational associations are supported by longitudinal studies, controlled interventions, causal-inference approaches or mechanistic experiments.

Funding and reporting practices also deserve attention when evaluating intervention studies. Industry involvement is common in some areas of probiotic research. For example, a systematic review of randomized trials of probiotic-, prebiotic- and synbiotic-supplemented infant formula found that 40 of 67 completed trials were funded by the food industry (Mugambi et al., 2013). However, industry sponsorship should not itself be treated as evidence of biased results: that review found no significant association between funding source and most clinical outcomes, and a later review of probiotic trials for acute diarrhoea likewise found no significant association between industry funding and positive outcomes (Saa et al., 2019). Funding source, sponsor involvement, conflicts of interest and methodological quality should therefore be considered when judging the strength of individual studies rather than assuming that sponsorship necessarily invalidates their findings.

A gap remains between the large number of reported microbiota–disease associations and the smaller number that have translated into reproducible clinical applications. Germ-free animals, antibiotic-treated models and microbiota-transfer experiments are valuable for testing biological mechanisms, but their findings cannot be assumed to apply directly to humans. Likewise, a treatment-associated change in microbial composition or metabolite levels does not by itself demonstrate clinical benefit or causal mediation. Preclinical mechanisms, observational human associations and replicated clinical effects should therefore be distinguished when evaluating evidence for microbiota–gut–brain mechanisms and therapies.

#### 5.1.2 Safety and regulatory bottlenecks

Safety considerations differ among microbiota-targeted interventions. Probiotics are generally well tolerated, but rare cases of invasive infection, including bacteremia and fungemia, have been reported, particularly in vulnerable patients (Costa et al., 2018). FMT presents different risks because donor material may transmit infectious agents, including multidrug-resistant organisms. Such events have prompted regulatory authorities to strengthen donor screening and stool-testing requirements (DeFilipp et al., 2019). Donor selection, pathogen screening and manufacturing controls are therefore important components of FMT safety (Goldsmith et al., 2024). Screening requirements may also need to change as new transmissible risks emerge, as illustrated by additional safety measures introduced during the SARS-CoV-2 pandemic (DeFilipp et al., 2019; Green et al., 2020).

Regulation of FMT remains heterogeneous across jurisdictions, with differences in product classification, clinical indications and regulatory oversight (Yadegar et al., 2024). These differences complicate the development of consistent approaches to donor eligibility, manufacturing, quality control and post-treatment surveillance. Probiotic products present different quality-control issues, including strain identification, viability and product quality (Binda et al., 2020). Dietary interventions raise different safety concerns: prolonged restrictive diets may compromise nutritional adequacy, while KD can cause gastrointestinal and metabolic adverse effects and may require clinical monitoring (Schopf et al., 2026). Pharmacological approaches should likewise be evaluated according to their established adverse-effect profiles rather than being considered safe simply because they may influence the gut microbiome. Safety assessment and regulatory oversight therefore need to be tailored to the characteristics and risks of individual gut–brain-axis interventions.

### 5.2 Future outlook

Improving reproducibility will require greater consistency in how microbiome studies are conducted and reported. For multicentre studies, predefined procedures for sample collection, storage, DNA extraction, sequencing and bioinformatic analysis would reduce avoidable technical variation, while medication use, diet and bowel transit should be recorded as potential sources of biological variation. Standardized procedures for faecal sample processing have already been proposed and may improve comparability across studies (Costea et al., 2017). Microbial signatures identified in one cohort should be tested in independent populations to see whether the same patterns can be reproduced and whether their diagnostic or predictive performance is maintained.

Cross-sectional studies cannot determine whether microbiome alterations contribute to disease or occur as a consequence of it. Longitudinal and interventional studies can help clarify these relationships, while findings from animal models need to be confirmed in humans before they can inform clinical practice (Metwaly et al., 2025).

Changes in the microbiome during treatment do not necessarily explain clinical improvement. Microbial changes may contribute to treatment response, but they may also occur as a consequence of treatment. Repeated measurements during treatment may help establish whether these changes precede or follow clinical improvement.

Multi-omics and machine-learning approaches may reveal microbial and metabolic patterns that are missed by taxonomic analyses alone. However, incomplete or inconsistent metadata can make it difficult to account for confounding factors and may reduce model reliability (Kumar et al., 2024). Before microbiome-based biomarkers can be considered for clinical use, the analytical methods used to measure them also need to be adequately validated (Rodriguez et al., 2025).

## 6 Conclusion

Current research supports a role for the gut microbiota in communication between the gastrointestinal tract and the brain through neural, endocrine, immune and metabolic pathways. Experimental studies have provided mechanistic evidence for several of these pathways, including signaling mediated by microbial metabolites and host immune responses. Their contribution to human disease, however, is less firmly established. Many microbial signatures associated with neurological, psychiatric and metabolic disorders have been identified in observational studies, making it difficult to determine whether they contribute to disease development, result from disease-related changes, or reflect other host and environmental factors.

Microbiota-targeted interventions have also produced mixed levels of evidence. Probiotics, prebiotics, synbiotics, dietary interventions, fecal microbiota transplantation and pharmacological approaches can alter microbial or metabolic features, and some have shown clinical effects in specific populations. These findings should not be taken to mean that changes in the microbiome necessarily account for the observed clinical response. Evidence from animal models and exploratory human studies remains useful for identifying possible mechanisms and therapeutic targets, but confirmation in well-designed and independently replicated clinical studies is still required for many applications.

The microbiota–gut–brain axis therefore provides a useful framework for studying interactions between intestinal microorganisms and host physiology, while its clinical relevance needs to be considered on a mechanism- and disease-specific basis. At present, the strongest conclusions are those supported by converging mechanistic and human evidence. Other proposed pathways, biomarkers and therapeutic applications remain promising but insufficiently validated for routine clinical use.

## Glossary

HPA axis: Hypothalamic–pituitary–adrenal axis
PD: Parkinson’s disease
BBB: Blood–brain barrier
CNS: Central nervous system
ENS: Enteric nervous system
SCFAs: Short-chain fatty acids
GABA: γ-aminobutyric acid
5-HT: 5-hydroxytryptamine
CCK: Cholecystokinin
NTS: Nucleus tractus solitarius
IL-6: Interleukin-6
TNF-α: Tumor necrosis factor-α
IBD: Inflammatory bowel disease
IBS: Irritable bowel syndrome
NE: Norepinephrine
α7nAChR: α7 nicotinic acetylcholine receptors
ACh: Acetylcholine
GLP-1: Glucagon-Like Peptide-1
PYY: Peptide YY
CRH: Corticotropin-releasing hormone
ACTH: Adrenocorticotropic hormone
TLRs: Toll like receptors
5-HTP: 5-hydroxytryptophan
IPA: 3-Indolepropionic acid
TPH1: Tryptophan hydroxylase 1
AhR: Aryl hydrocarbon receptor
IDO: Indoleamine-2,3-dioxygenase
TDO: Tryptophan-2,3-dioxygenase
AD: Alzheimer’s disease
Aβ: Amyloid-β
ITF: Inulin-type fructans
Tregs: Regulatory T cells
FMT: Fecal Microbiota Transplantation
MDD: Major depressive disorder
Med Diet: Mediterranean Diet
Low FODMAP diet: Low fermentable oligosaccharides, disaccharides, monosaccharides, and polyols diet
KD: Ketogenic Diet
ESC: Escitalopram
CBT: Cognitive Behavioral Therapy
SSRI: Selective serotonin reuptake inhibitor
I3PA: Indole-3-propionic acid
NF-κB: Nuclear factor-κB
TGR5: Takeda G-protein-coupled receptor 5
FXR: Farnesoid X receptor
IL-1β: Interleukin-1β
rCDI: recurrent *Clostridioides difficile* infection
TCM: Traditional Chinese Medicine
HBD2: Human β-defensin 2
