# Supplementary material for: Targeting the gut–brain axis: novel strategies for disease intervention from mechanisms to clinical practice
Source: Front Microbiol. 2026 Oct 7;17:1923456. doi: 10.3389/fmicb.2026.1923456 (PMC13684330; doi:10.3389/fmicb.2026.1923456)
Supplement: Supplementary file 1 [file Table_1.DOCX]

**Table 1 Representative Probiotic Strains in Gut–Brain Axis Research**

| Probiotic strains | Study type | Sample size | Dose | Main Functions | Major outcomes | Level evidence | Reference |
| --- | --- | --- | --- | --- | --- | --- | --- |
| *Lactobacillus helveticus R0052 and Bifidobacterium longum R0175* | Human RCT | n=55 | 3×10⁹ CFU/day, 30 days | Reduce scores of depression, somatisation and anger‑hostility sub‑scales | Reduced several psychological symptom scores, including somatization, depression and anger-hostility subscales | Low–Moderate | doi:10.1016/j.jad.2019.04.043 |
| *Lactobacillus casei* | Human RCT | n=47 | 100×10⁹ CFU/day, 8 weeks | Maintain gut microbiota diversity and relieve stress-induced gastrointestinal symptoms | Preserved gut microbial diversity and reduced stress-associated abdominal symptoms | Low–Moderate | doi:10.3920/BM2015.0100 |
