# Supplementary material for: Targeting the gut–brain axis: novel strategies for disease intervention from mechanisms to clinical practice
Source: Front Microbiol. 2026 Oct 7;17:1923456. doi: 10.3389/fmicb.2026.1923456 (PMC13684330; doi:10.3389/fmicb.2026.1923456)
Supplement: Supplementary file 2 [file Table_2.DOCX]

**Table 2. Representative studies of dietary interventions relevant to the microbiota–gut–brain axis**

| Dietary intervention | Study type | Population/model and sample size | Intervention and duration | Major outcomes | Evidence and limitations | Representative study |
| --- | --- | --- | --- | --- | --- | --- |
| Med Diet | Cross-sectional human study | 153 healthy adults following omnivorous, vegetarian, or vegan diets | Habitual dietary intake; Mediterranean diet adherence assessed rather than experimentally assigned | Higher adherence to a Mediterranean-style diet was associated with higher fecal SCFAs and differences in fiber-associated microbial taxa; lower adherence was associated with higher urinary trimethylamine oxide | Provides human microbiome and metabolomic evidence, but the cross-sectional design does not establish causality | doi:10.1136/gutjnl-2015-309957 |
| Med Diet | Multicenter dietary intervention | 612 non-frail or pre-frail older adults from five European countries with paired microbiome samples | Individually tailored Mediterranean diet for 12 months | Diet adherence was associated with microbiome changes; diet-responsive taxa were associated with lower frailty, better cognitive measures, and lower inflammatory markers | Large multicenter human study with longitudinal microbiome data; associations between microbial changes and health outcomes do not prove mediation | doi:10.1136/gutjnl-2019-319654 |
| Med Diet | Randomized clinical trial | 447 older adults at high cardiovascular risk | Mediterranean diet supplemented with extra-virgin olive oil or nuts versus a control diet; mean follow-up about 4.1 years | Mediterranean diet groups showed more favorable changes in cognitive performance than the control group | Provides direct clinical evidence for cognitive outcomes, but gut microbiota was not tested as the mediator of benefit | doi:10.1001/jamainternmed.2015.1668 |
| Low FODMAP diet | Randomized, controlled crossover trial | 30 adults with IBS | Controlled low-FODMAP diet versus a typical Australian diet; 3 weeks per dietary period | Gastrointestinal symptoms, including overall symptoms and bloating, were lower during the low-FODMAP period | Direct short-term RCT evidence for IBS symptom control; small sample and short intervention limit conclusions about long-term effects | doi:10.1053/j.gastro.2013.09.046 |
| Low FODMAP diet | Systematic review and meta-analysis | 12 intervention studies; 772 participants | Low-FODMAP interventions ranging from 4 days to 3 months | Low-FODMAP diets reduced IBS symptom severity and improved quality of life compared with control diets | Supports clinical efficacy across trials, although study designs, control diets, intervention duration, and outcomes were heterogeneous | doi:10.1007/s00394-020-02473-0 |
| KD | Prospective clinical microbiome study | 12 children receiving KD; 11 untreated parents served as diet controls | Classic KD for 3 months; ketogenic ratio 3:1–4:1 | KD altered both taxonomic and functional gut microbiota profiles; five of 12 children had >50% seizure reduction | Direct human microbiome evidence, but the cohort was very small and microbial changes were not shown to mediate seizure control | doi:10.1038/s41522-018-0073-2 |
| KD | Systematic review of randomized controlled trials | 14 RCTs involving 1,114 children and adolescents | Various ketogenic dietary protocols; most seizure outcomes assessed after approximately 3–4 months | Six of 14 trials reported significantly greater >50% seizure reduction in KD-treated groups; gastrointestinal symptoms were the most frequent adverse events | Supports clinical efficacy in pediatric drug-resistant epilepsy; does not establish that gut microbiota is the main mechanism of benefit | doi:10.1007/s13668-022-00405-4 |
| KD | In vivo mechanistic study | Two mouse seizure models; group sizes varied across experiments | KD feeding combined with germ-free, antibiotic-treatment, bacterial colonization, and microbiota-transfer experiments | KD-induced seizure protection was lost after microbiota depletion and restored by selected KD-associated bacteria, including *Akkermansia* and *Parabacteroides* | Strong mechanistic evidence in animals, but microbiota-mediated seizure protection has not been demonstrated directly in patients | doi:10.1016/j.cell.2018.04.027 |
